# Hydroethanolic Extract of *Prunus domestica* L.: Metabolite Profiling and In Vitro Modulation of Molecular Mechanisms Associated to Cardiometabolic Diseases

**DOI:** 10.3390/nu14020340

**Published:** 2022-01-14

**Authors:** Hammad Ullah, Eduardo Sommella, Cristina Santarcangelo, Danilo D’Avino, Antonietta Rossi, Marco Dacrema, Alessandro Di Minno, Giacomo Di Matteo, Luisa Mannina, Pietro Campiglia, Paolo Magni, Maria Daglia

**Affiliations:** 1 Department of Pharmacy, University of Napoli Federico II, Via D. Montesano 49, 80131 Naples, NA, Italy; hammad.ullah@unina.it (H.U.); cristina.santarcangelo@unina.it (C.S.); dani.davino@studenti.unina.it (D.D.); antrossi@unina.it (A.R.); marco.dacrema@unina.it (M.D.); alessandro.diminno@unina.it (A.D.M.); 2 Department of Pharmacy, University of Salerno, 84084 Fisciano, SA, Italy; esommella@unisa.it (E.S.); pcampiglia@unisa.it (P.C.); 3 CEINGE-Biotecnologie Avanzate, Via Gaetano Salvatore 486, 80145 Naples, NA, Italy; 4 Department of Chemistry and Technology of Drugs, Sapienza University of Rome, Piazzale Aldo Moro 5, 00185 Rome, RM, Italy; giacomo.dimatteo@uniroma1.it (G.D.M.); luisa.mannina@uniroma1.it (L.M.); 5 European Biomedical Research Institute of Salerno, Via De Renzi 50, 84125 Salerno, SA, Italy; 6 Department of Pharmacological and Biomolecular Sciences, Università degli Studi di Milano, 20133 Milan, MI, Italy; 7 IRCCS MultiMedica, Sesto San Giovanni, 20099 Milan, MI, Italy; 8 International Research Center for Food Nutrition and Safety, Jiangsu University, Zhenjiang 212013, China

**Keywords:** *Prunus domestica* L., chemical characterization, digestive enzyme inhibition, HMG-CoA reductase inhibition, anti-inflammatory activity

## Abstract

High consumption of fruit and vegetables has an inverse association with cardiometabolic risk factors. This study aimed to chemically characterize the hydroethanolic extract of *P. domestica* subsp. syriaca fruit pulp and evaluate its inhibitory activity against metabolic enzymes and production of proinflammatory mediators. Ultra-high-performance liquid chromatography high-resolution mass spectrometry(UHPLC-HRMS) analysis showed the presence of hydroxycinnamic acids, flavanols, and glycoside flavonols, while nuclear magnetic resonance(NMR) analysis showed, among saccharides, an abundant presence of glucose. *P. domestica* fruit extract inhibited α-amylase, α-glucosidase, pancreatic lipase, and HMG CoA reductase enzyme activities, with IC50 values of 7.01 mg/mL, 6.4 mg/mL, 6.0 mg/mL, and 2.5 mg/mL, respectively. *P. domestica* fruit extract inhibited lipopolysaccharide-induced production of nitrite, interleukin-1 β and PGE_2_ in activated J774 macrophages. The findings of the present study indicate that *P. domestica* fruit extracts positively modulate in vitro a series of molecular mechanisms involved in the pathophysiology of cardiometabolic diseases. Further research is necessary to better characterize these properties and their potential application for human health.

## 1. Introduction

The metabolic syndrome (MS), (also called as “Reaven’s syndrome”, “Syndrome X”, “Insulin resistance syndrome”, and “the deadly quartet”) [1,2,3], is a cluster of pathological conditions including visceral obesity, hyperglycemia, or type 2 diabetes mellitus (T2DM), hypertension, and dyslipidemia, and is associated with higher cardiovascular disease (CVD) risk. A prolonged and persistent condition of MS may indeed silently progress towards serious complications such as T2DM, when not yet present, coronary heart disease (CHD), heart failure, stroke, cerebrovascular accidents (CVA) and stroke, as well as liver complications (nonalcoholic fatty liver disease (NAFLD) and nonalcoholic steatohepatitis (NASH)) [4,5]. Adipose tissue dysfunction is commonly associated with MS, and dysregulated secretion of pro- or anti-inflammatory adipokines may contribute towards MS-induced complications [6,7]. Indeed, elevation of circulating tumor necrosis factor-alpha (TNF-α), interleukin-6 (IL-6), and C-reactive protein (CRP) are predominantly observed in patients with dysregulated metabolism [8,9].

Increasing age, sedentary lifestyle, female gender, lower socioeconomic class, positive family history, excessive alcohol intake, unhealthy dietary patterns, and intake of numerous medications are some of the risk factors associated with the prevalence of MS [10,11]. In this context, prevention is essential. Adopting a healthy lifestyle, aimed at weight normalization, increasing physical activity and adoption of a healthy diet, including increased intake of fruits, vegetables, and whole grains and a low consumption of salt, trans fatty acids, and cholesterol-rich foods [12]. However, as maintaining a healthy lifestyle on a daily basis is challenging, specific pharmacological interventions (i.e., statins, renin–angiotensin–aldosterone system (RAAS) inhibitors, and insulin-sensitizing agents, etc.) are often prescribed to target some selected pathogenetic mechanisms [12,13,14]. The main limitations of the pharmacological approach are the increase in the occurrence of adverse and side effects without beneficial effects on low-grade inflammation [15]. In this context, there is a growing attention to food supplements able to decrease the modifiable risk factors of MS [16]. One of the most widely used food supplement ingredients is red yeast rice (RYR), made by fermentation of rice with *Monascus purpureus*, producing monacolin K, which, in lactone form, is identical to lovastatin. RYR is used alone or in combination with plant extracts, coenzyme Q10, chromium, and vitamins. In 2018, the Panel on Food Additives and Nutrient Sources added to Food of the European Food Safety Authority (EFSA) has expressed doubts about the actual safety of RYR and concluded that EFSA is “unable to identify a dietary intake of monacolins from RYR that does not give rise to concerns about harmful effects to health” [17]. Besides RYR, other natural compounds could be effective strategies in the treatment of MS and prevention of MS progression towards serious complications through the inhibitory activity of the enzymes associated with carbohydrate/lipid digestion and cardiometabolic diseases (i.e., α-amylase, α-glucosidase, HMG-CoA reductase, and pancreatic lipase) [18,19,20].

Research analysis has shown an inverse association of increased consumption of fruits and vegetables with the incidence of MS risk factors [21,22], though there is a strong need to identify the specific effects of fruits and vegetables on the risk of MS development [23]. *Prunus domestica* L. (European plum) is known for its health benefits, which may be the result of its antioxidant potential and anti-inflammatory effects [24,25]. Plums are fruits with low glycemic index, thus their consumption in adequate amount and on a regular basis could be a potential preventive strategy against MS [25,26]. In addition, plums are commonly consumed when on a diet, they are commercially available at low cost, and as happens for many fruits, some of the plums produced are discarded as they do not reach the size requirements to be placed on the market.

As finding new food supplement ingredients which are safe and effective in the prevention of MS is an unmet need, especially after the concern of EFSA regarding RYR, the present study aimed to (1) characterize the metabolite profile of a hydroethanolic extract of *Prunus domestica*, using a multimethodological approach that was previously used for the study of different food matrices [27,28], and two analytical techniques, untargeted nuclear magnetic resonance (NMR) spectroscopy and targeted ultra-high-performance liquid chromatography high-resolution mass spectrometry (UHPLC-HRMS), and (2) study the in vitro modulatory effects of this extract on the activity of a panel of enzymes differentially involved in the pathophysiology of MS and the modulation of proinflammatory mediator release.

## 2. Materials and Methods

### 2.1. Preparation of Fruit Extracts

**Figure 1:**
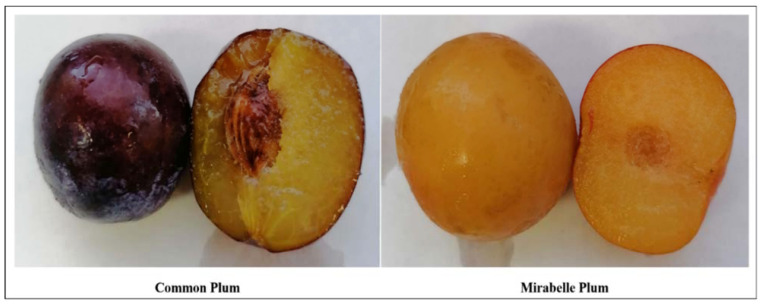
Plum varieties, collected from a local cultivator in the Campania Region (Italy). *P. domestica* L. subsp. domestica (Common plum) and *P. domestica* L. subsp. syriaca (Mirabelle plum).

Two different varieties of *Prunus domestica* L. fruits (*P. domestica* L. subsp. domestica, also known as common plum, and *P. domestica* L. subsp. syriaca, also known as Mirabelle plum) (Figure 1) were collected from a local cultivator in the Campania Region (Italy) in October 2020. Eight fruits were sampled for each variety (Common Plum and Mirabelle Plum). All fruits were first washed with water to eliminate every dirt residue and were separated into skin and pulp. Both parts were cut into small pieces with a ceramic knife. To control oxidation during preparation, the samples were cut in an ice bath. The samples were freeze-dried and then ground into fine powder using a mortar and pestle. Aliquots of 1 and 2 g of the powdered skin and pulp were added to 20 mL and 40 mL of 50%, 70% and 99% ethanolic solution acidified with 0.1% HCL solution, respectively. The sample pH values were adjusted to 2.0, and the samples were subjected to magnetic stirring for 3 h at room temperature, followed by centrifugation at 6000 rpm for 10 min. The precipitate was separated from the supernatant. The same procedure was repeated three times and the supernatants of each sample were collected and filtered through Whatman cellulose filter paper. The filtrate was concentrated in a rotary evaporator at a temperature lower than 30 °C and submitted to freeze drying. The dry extracts were kept at −20 °C for subsequent determination of total polyphenol content, antioxidant activity, untargeted NMR spectroscopy, and targeted UHPLC-HRMS.

Based on the assessment of total polyphenol content and antioxidant activity, the fruit pulp extract of *P. domestica* subsp. syriaca obtained with 50% hydroethanolic solution was selected, and in view of the high content of glucose and sucrose determined via NMR, it was subjected to the chemical precipitation of its sugars by treatment with absolute ethanol, followed by an ultra-freezing temperature. The organic solvent was removed under reduced pressure by a rotary evaporator and the dry extract obtained from the fruit pulp extract of *P. domestica* subsp. syriaca without sugars was kept at −20 °C for subsequent biological assays.

### 2.2. Total Phenolic Contents

Total phenolic content (TPC) was determined using a colorimetric assay (Folin-Ciocalteu method), following the same protocol as set by Singleton et al. with some modifications [29]. An aliquot (10 µL) of the samples (50 mg/mL) or gallic acid standard solutions (200–1000 µg/mL) was taken and added to 50 µL of Folin-Ciocalteu reagent. The solutions were cyclomixed for 4 min and added to 200 µL Na_2_CO_3_ (15%). The final volume was made to 1 mL with distilled water and allowed to incubate for 2 h at room temperature and under dark conditions. The solutions were read spectrophotometrically at 750 nm. Gallic acid was used as standard compound, with serial dilutions being prepared with known concentrations ranging from 200 to 1000 µg/mL. The results were expressed as mg equivalent to gallic acid/g of extract on dry weight basis.

### 2.3. Antioxidant Assay

ABTS (2,2′-azino-bis3-ethylbenzthiazoline-6- sulfonic) assay was performed to evaluate the antioxidant potential of the fruit extracts following protocols set by Kok et al. with slight modifications [30]. The assay was conducted by placing 1 mL ABTS solution in a microtube, to which 10 µL of sample (50 mg/mL) or Trolox (0, 15, 20, 25, 30 and 35 µM) was added. The mixture was allowed to incubate for 2.5 min, and the absorbance was read at 734 nm. Thus, the antioxidant compounds present in the fruit extracts quench the color and produce a decoloration of the solution which is proportional to their antioxidant activity. The results were expressed as Trolox equivalent concentration (µM/g of extract on dry weight basis).

### 2.4. Metabolic Profiling of P. domestica Fruit Pulp Extract

The metabolic profiling of *P. domestica* fruit pulp extract was evaluated using UHPLC-HRMS and NMR analysis.

#### 2.4.1. RP-UHPLC-HRMS Analysis

*P. domestica* fruit pulp extract was solubilized in methanol/water (50:50 *v/v*). The sample was then filtered through a cellulose acetate/cellulose nitrate mixed esters membrane (0.45 μm; Millipore Corporation, Billerica, MA, USA), and analyzed by RP-UHPLC-HRMS. UHPLC-HRMS analysis was performed on a Shimadzu Nexera UHPLC system, consisting of a CBM-20A controller, two LC-30AD dual-plunger parallel-flow pumps, a DGU-20 AR5 degasser, an SPD-M20A photo diode array detector (PDA), a CTO-20A column oven, and a SIL-30AC autosampler. The system was coupled online to a hybrid Ion trap Time of Flight Mass spectrometer (LCMS-IT-TOF, Shimadzu, Duisburg, F.R. Germany) equipped with an electrospray source (ESI). For RP-UHPLC analysis, a Kinetex Biphenyl 100 mm × 2.1 mm, 2.6 µm (L × I.D, particle size, Phenomenex^®^, Bologna, Italy) column was employed at a flow rate of 0.4 mL/min. The mobile phases consisted of (A) 0.1% CH_3_COOH in H_2_O and (B) ACN plus 0.1% CH_3_COOH. Analysis was performed in gradient as follows: 0–20.0 min, 2–20% B; 20.01–22.0 min, 20.01–99% B; 99% B hold for 1 min; returning to initial conditions in 0.1 min. The column oven was set to 40 °C and 5 µL sample was injected. PDA detection parameters were sampling rate 12 Hz, time constant 0.160 s and chromatograms were extracted at 280 and 330 nm. LC data elaboration was performed by the LCMS solution^®^ software (Version 3.50.346, Shimadzu, Duisburg, F.R. Germany). MS detection was performed in negative mode ionization as follows: curve desolvation line (CDL), 250 °C; Block Heater, 250 °C; Nebulizing and Drying gas, 1.5 and 10 L/min; ESI^-^ Capillary Voltage, −3.5 kV; MS range, m/z 150–1500; ion accumulation time, 30 ms; ion trap repeat, 3. MS/MS was performed in a data-dependent acquisition (DDA), precursor ions selection was based on the base peak chromatogram (BPC) intensity of 700,000. Collision-induced dissociation (CID), 50%, ion trap repeat. For analysis, the instrument was tuned using sodium trifluoroacetate (NaTFA). Metabolite annotation was based on accurate mass measurement, MS/MS fragmentation pattern and comparison within silico spectra with MS database searching [31,32]. “Formula Predictor” software (Shimadzu, Duisburg, F.R. Germany) was used for the prediction of the molecular formula using the following settings: maximum deviation from mass accuracy: 5 ppm, fragment ion information, and nitrogen rule.

#### 2.4.2. NMR Analysis

*P. domestica* fruit pulp extract (500 mg) was solubilized in 10 mL of 400 mM phosphate buffer/D2O containing 3-(trimethylsilyl)-propionic-2,2,3,3-d4 acid sodium salt (TSPA), used as internal standard for quantitative measurements, and EDTA, used as a complexing agent for metal ions. Then, an aliquot of 0.7 mL was transferred in a 5 mm NMR tube. The NMR spectra were recorded at 25 °C on a JNM-ECZ 600R (JEOL Ltd., Tokyo, Japan) spectrometer operating at the proton frequency of 600.17 MHz equipped with an autosampler and the SuperCOOL cryogenic probe (JEOL Ltd., Tokyo, Japan). The ^1^H spectrum was acquired using a presaturation pulse sequence to suppress water signal, a 90° pulse of 12.8 μs and 65 K data points. All the NMR spectra were processed using the JEOL Delta v5.3.1. software (JEOL Ltd., Tokyo, Japan). The ^1^H spectrum after Fourier transformation was manually phased, automatically base-corrected and referred to the β-glucose CH-1 signal set at 4.66 ppm. 2D NMR experiments, namely ^1^H-^1^H COSY, ^1^H-^1^H TOCSY,^1^H-^13^C HSQC and ^1^H-13C HMBC, were performed using the following experimental conditions: ^1^H-^1^H COSY and ^1^H-^1^H TOCSY experiments were carried out with water presaturation during relaxation delay and 9 kHz of spectral width in both dimensions. ^1^H-^1^H COSY was acquired using 4 k × 256 points in F1 and F2, respectively, a relaxation delay of 2.5 s and 44 scans, whereas in the case of ^1^H-^1^H TOCSY experiment, 8 k × 256 points in F1 and F2 dimensions, respectively, a mixing time of 80 ms, a relaxation delay of 2 s and 52 scans were used. ^1^H-^13^C HSQC experiment was carried out using a 90° ^1^H pulse of 12.8 μs and 90° ^13^C pulse of 14.0 μs, a spectral width of 9 kHz and 33 kHz for the ^1^H and ^13^C dimensions, respectively, 8 k × 256 points, a relaxation delay of 2 s, 80 scans and a coupling constant 1JC–H of 150 Hz. ^13^C spectra were referenced to the CH-1 resonance of β-glucose at 97.00 ppm. ^1^H-13C HMBC experiment was carried out with 12.8 μs for ^1^H and 14.0 μs for ^13^C 90° pulse, a spectral width of 9 kHz and 38 kHz for the ^1^H and ^13^C dimensions, respectively, 8 k × 256 points in F1 and F2 dimensions, a relaxation delay of 2 s, a delay for the evolution of long-range couplings of 50 ms and 76 scans. In order to quantify the assigned compounds, the integral of the corresponding selected ^1^H resonances were measured with respect to the integral of TSP methyl group signal normalized to 100. Quantitative results were expressed in μg/mg of dry weight.

### 2.5. Enzyme Inhibition Assays

Inhibition assays of different enzymes associated with the MS were performed as described below. The selected fruit extract (*P. domestica* subsp. syriaca fruit pulp), dissolved in 1% DMSO (SERVA Electrophoresis GmbH, Aurogene, Rome, Italy) and respective positive controls were tested using different concentrations to obtain half minimal inhibitory concentration (IC_50_) for each enzyme, by nonlinear regression analysis. The absorbance of the sample blank (buffer in place of enzyme solution) and control (buffer in place of extract) was recorded as well. The inhibition of enzyme activity was calculated using following equation:

% inhibition = [(A _control_ − A _extract_)/A _control_] × 100

#### 2.5.1. α-Amylase Inhibition Assay

The α-amylase from porcine pancreas inhibition assay was performed according to the protocol set up by Cicolari et al. with slight modifications [33]. The reaction mixture contained 20 µL fruit extract solution (concentration range: 0.0625–25 mg/mL) or acarbose (concentration range: 15.56–400 µg/mL), and 20 µL enzyme solution (0.5 mg/mL) in 0.02 M sodium phosphate buffer (pH 6.9 with 0.006 M NaCl), which was preincubated for 10 min at 25 °C. Then, 20 µL of 1% starch solution was added to each tube at timed intervals and allowed to incubate for 10 min at 25 °C. The reaction was stopped by the addition of 40 µL color reagent (DNSA). The test tubes were incubated in a boiling water bath for 10 min, and then cooled to room temperature. Finally, 600 µL of bidistilled water was added to dilute the reaction mixture and the absorbance was read at 540 nm using a microplate reader.

#### 2.5.2. α-Glucosidase Inhibition Assay

The α-glucosidase from *Saccharomyces cerevisiae* inhibition assay was performed according to the protocols set by Cicolari et al. with slight modifications [33]. The reaction mixture containing 50 µL fruit extract solution (concentration range: 0.0313–25 mg/mL) or acarbose (concentration range: 15.56–900 µg/mL), and 100 µL enzyme solution (1 unit/mL) in 0.1 M phosphate buffer (pH 6.9), was incubated in a 96-well plate for 10 min at 25 °C. After preincubation, 50 µL of 0.1 M phosphate buffer (pH 6.9) solution containing 5 mM p-nitrophenyl-α-D-glucopyranoside was added to each well at timed intervals, and was incubated for 5 min at 25 °C. The absorbance was read at 405 nm using a microplate reader.

#### 2.5.3. HMG-CoA Reductase Inhibition Assay

The assay was conducted according to the manufacturer’s protocol (Sigma-Aldrich). The assay was conducted by placing 910 μL phosphate buffer with 5 μL fruit extract (concentration range: 0.0625–30 mg/mL) or 5 µL pravastatin (concentration range: 18.75–300 µM) into microtubes; 20 μL of NADPH and 60 μL of HMG-CoA reductase substrate were then added. The analysis was initiated (time 0) by the addition of 5 μL of HMG-CoA reductase, and incubated at 37 °C. The rate of NADPH consumed was monitored every 15 s for up to 5 min by reading the decrease in absorbance at 340 nm, using the microplate reader.

#### 2.5.4. Pancreatic Lipase Inhibition Assay

Porcine Pancreatic Lipase (PPL) inhibition assay was conducted according to the protocols reported by Nwakiban et al. (2019) [34]. The assay was conducted by mixing 30 μL PPL (2.5 mg/mL in 10 mM MOPS and 1 mM EDTA, pH 6.8) with 850 μL Tris buffer (100 mM Tris-HCl and 5 mM CaCl2, pH 7.0). Then, either 100 μL of fruit extract (concentration range: 1.30–12.5 mg/mL) or orlistat (concentration range: 1–500 µg/mL) was added to the mixture and incubated for at 37 °C for 15 min, followed by the addition of 10 μL substrate (10 mM p-NPB in dimethyl formamide). The mixtures were incubated again at 37 °C for 30 min. The absorbance was read at 405 nm using a microplate reader, to determine the lipase activity by quantifying the hydrolysis of p-NPB to p-nitrophenol.

### 2.6. Cell Culture

Murine monocyte/macrophage J774 cell line was obtained from the American Type Culture Collection (ATTC TIB 67). The cell line was grown in adhesion in Dulbecco’s modified Eagles medium (DMEM) supplemented with glutamine (2 mM, Aurogene Rome, Italy) Hepes (25 mM, Aurogene Rome, Italy) penicillin (100 U/mL, Aurogene Rome, Italy), streptomycin (100 μg/mL, Aurogene Rome, Italy), fetal bovine serum (FBS, 10%, Aurogene Rome, Italy) and sodium pyruvate (1.2%, Aurogene Rome, Italy) (DMEM completed). The cells were plated at a density of ~1 × 10^6^ cells in 75 cm^2^ culture flasks and maintained at 37 °C under 5% CO_2_ in a humidified incubator until 90% confluence. The culture medium was changed every 2 days. Before a confluent monolayer appeared, sub-culturing cell process was carried out. *P. domestica* subsp. syriaca fruit pulp extract was solubilized in DMSO at the concentration of 200 mg/mL (stock solution). Then, it was diluted in DMSO to obtain solutions at the concentrations of 150 mg/mL, 100 mg/mL, 20 mg/mL and 2 mg/mL. Cells were plated to a seeding density of 5.0 × 10^5^ in 24 multiwell plates. After 2 h of adhesion, cells were pretreated (for 2 h) with increasing concentration of *P. domestica* subsp. syriaca fruit pulp extract (5 µL of 2, 20, 100, 150 and 200 mg/mL, which correspond to a final concentration in the well (1 mL) of 0.01, 0.1, 0.5, 0.75 and 1 mg/mL). After the preincubation, macrophages were stimulated with or without LPS from *Escherichia coli*, Serotype 0111:B4, (10 μg/mL; 100 µL of solution 100 μg/mL in DMEM completed with FBS, Sigma Aldrich, Milan, Italy) for 24 h [35].

#### 2.6.1. Nitrite, IL-1β and PGE_2_ Assay

After 24 h of incubation, the supernatants were collected for the nitrite, IL-1β and PGE_2_ measurement. The nitrite concentration in the samples was measured by the Griess reaction, by adding 100 μL of Griess reagent (0.1% naphthylethylenediamide dihydrochloride in H_2_O and 1% sulphanilamide in 5% concentrated H_2_PO_4_; vol. 1:1; Sigma Aldrich, Milan, Italy) to 100 μL samples. The optical density at 540 nm (OD_540_) was measured immediately after Griess reagent addition, using ELISA microplate reader (Thermo Scientific, Multiskan GO, Milan Italy). Nitrite concentration was calculated by comparison with OD_540_ of standard solutions of sodium nitrite prepared in culture medium. IL-1β (R&D Systems, Aurogene, Rome, Italy) and PGE_2_ (Cayman Chemical, BertinPharma, Montigny Le Bretonneux, France) levels were measured with commercially available ELISA kits according to the manufacturer’s instructions.

#### 2.6.2. Cell Viability

Cell respiration, an indicator of cell viability, was assessed by the mitochondrial-dependent reduction of 3-(4,5-dimethylthiazol-2-yl)-2,5-diphenyltetrazolium bromide (MTT; Sigma Aldrich, Milan, Italy) to formazan. Cells were plated to a seeding density of 1.0 × 10^5^ in 96 multiwell plates. After stimulation with LPS in the absence or presence of test compounds for 24 h, cells were incubated in 96-well plates with MTT (0.2 mg/mL), for 1 h. Culture medium was removed by aspiration and the cells were lysed in DMSO (0.1 mL). The extent of reduction of MTT to formazan within cells was quantified by the measurement of OD_550_.

### 2.7. Statistical Analysis

Results from at least three independent experiments carried out in triplicate for TPC and antioxidant assay, and two independent experiments carried out in duplicate for enzyme inhibitory activities of the *P. domestica* fruit extract, were expressed as mean (±SD) values. Student’s t-test was used to determine the level of significance and statistical differences among variables using GraphPad prism. For the in vitro anti-inflammatory studies, the results were expressed as mean ± standard error (SEM) of the mean of *n* observations, where *n* represents the number of experiments performed in different days. Triplicate wells were used for the various treatment conditions. The results were analyzed by one-way ANOVA followed by a Bonferroni post hoc test for multiple comparisons. A *p*-value less than 0.05 was considered significant. All graphs were generated using GraphPad prism, version 5 (GraphPad, San Diego, CA, USA).

## 3. Results

### 3.1. Description of P. domestica Extracts

Two different varieties of *Prunus domestica* L. fruits (*P. domestica* subsp. domestica and *P. domestica* subsp. syriaca), which were separated into pulp and skin, were submitted to three different hydroethanolic extractions to evaluate the effects of ethanol percentage in the extraction solvent on the total polyphenol content and antioxidant activity. The extraction yield calculated for the freeze-dried fruit skin ranged from 37 to 43% regardless of the *Prunus* variety. On the contrary, the extraction yield calculated for the freeze-dried fruit pulp ranged from 59.5 to 67% and from 45 to 49%, for subsp. domestica and subsp. syriaca, respectively (Table 1).

### 3.2. Total Phenolic Contents and In Vitro Antioxidant Activity

A total of 12 hydroethanolic extracts were evaluated for their total phenolic content (TPC) and in vitro antioxidant activity (Table 2). The pulp of *P. domestica* subsp. syriaca extracted with 50% ethanol showed the highest TPC (12.9 mg GAE/g on dry weight basis), followed by fruit skin of *P. domestica* L. subsp. domestica extracted with 70% ethanol containing 12.8 mg GAE/g on dry weight basis. Overall, the fruit extracts extracted with 99% of ethanol exhibited relatively less phenolics, with fruit pulp of *P. domestica* subsp. syriaca showing the lowest content of total phenolics (6.5 mg GAE/g on dry weight basis). In general, fruit skin showed the highest antioxidant activity with fruit skin of *P. domestica* L. subsp. domestica (extracted with 50% ethanol) showing a Trolox equivalent concentration of 1944.1 µM/g, while fruit pulp of *P. domestica* subsp. syriaca (extracted with 99% ethanol) showed the lowest Trolox equivalent concentration of 585.5 µM/g. On the basis of the higher polyphenol content, to which the inhibitory activity of the enzymes involved in MS is generally ascribed [29], the good antioxidant activity, the higher yield (49%), and the lower percentage of ethanol used as extraction solvent (50%), the *P. domestica* subsp. syriaca fruit pulp extract obtained with 50% hydroethanolic solution was selected for the subsequent chemical characterization.

Based on the assessment of total polyphenol content and antioxidant activity, on the higher yield (49%), and the lower percentage of ethanol used as extraction solvent (50%), the fruit pulp extract of *P. domestica* subsp. syriaca obtained with 50% hydroethanolic solution was selected, and in view of the high content of glucose and sucrose determined via NMR, it was subjected to the chemical precipitation of sugars by treatment with absolute ethanol, followed by ultra-freezing temperature. The organic solvent was removed under reduced pressure by a rotary evaporator, and the dry extract obtained from the fruit pulp extract of *P. domestica* subsp. syriaca without sugars was kept at −20 °C for subsequent biological assays.

### 3.3. UHPLC-HRMS Profile

**Figure 2:**
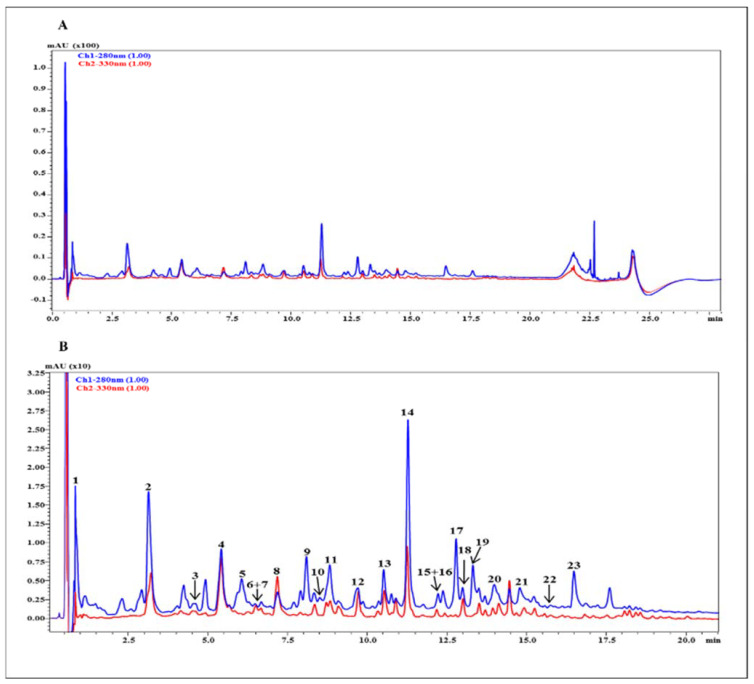
RP-UHPLC chromatograms of *P. domestica* subsp. syriaca fruit pulp extract with UV detection registered at λ 280 nm and 330 nm (**A**), and chromatogram expansion with corresponding peak HRMS assignment (**B**).

The list of the metabolites occurring in the hydroethanolic (50%) extract obtained from *P. domestica* subsp. syriaca fruit pulp, with tentative identification based on accurate mass and fragmentation pattern compared against reference MS/MS spectra reported in silico and in previous literature, is reported in Table 3. In particular, 23 compounds belonging to different classes (organic and hydroxycinnamic acids and flavonoids, both aglycone and glycosylated) were identified in the extract (Figure 2). Hydroxycinnamic and quinic acid derivatives were the most abundant compounds, retaining the 46.7% of total peak area, followed by procyanidins, in particular dimer (17%), monomers (13.10%) and trimers (6.9%). Lastly, flavonol glycosides represented the remaining 7.9%. (Table 4)

### 3.4. NMR Analysis and Quantification of Sugar and Organic Acid Contents

**Figure 3:**
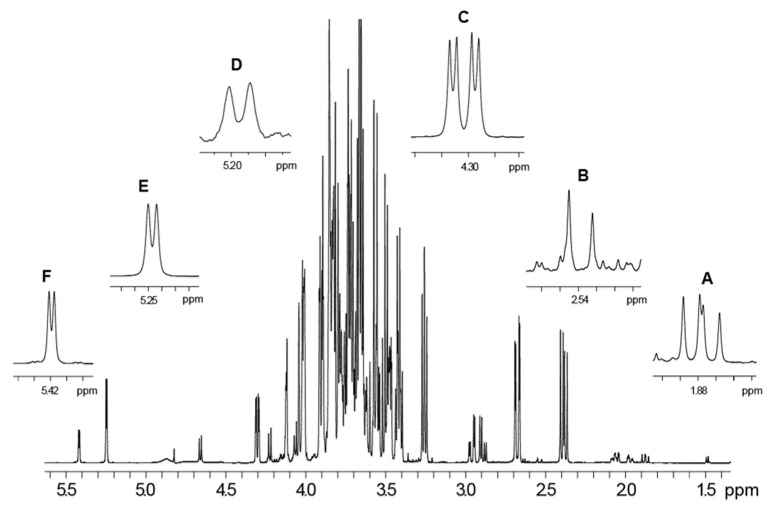
^1^H NMR spectrum of *P. domestica* subsp. syriaca fruit pulp extract. Quantified selected NMR signals are reported in expanded regions. (**A**) CH2-1 protons of quinic acid (1.88 ppm), (**B**) α,γ-CH protons of citric acid (2.54 ppm), (**C**) α-CH proton of malic acid (4.30 ppm), (**D**) CH-1 proton of in α-xylose (5.20 ppm), (**E**) CH-1 proton of α-glucose (5.25 ppm), (**F**) CH-1 proton of sucrose (5.42 ppm).

The ^1^H spectrum of the *P. domestica* subsp. syriaca fruit pulp extract dissolved in phosphate buffer/D2O shows the presence of glucose, sucrose, xylose and citric, malic and quinic acids. The ^1^H spectral assignment was obtained by literature data regarding other fruits [36,37] and 2D NMR experiments [38]. The integrals of selected signals due to sugars, namely xylose, glucose, and sucrose at 5.20 ppm, 5.25 ppm, and 5.42 ppm, respectively, and to organic acids, namely citric, malic and quinic acids at 1.88 ppm, 2.54 ppm, and 4.30 ppm, respectively, were used for compound quantification (Table 5). The ^1^H NMR spectrum with the selected signals used for the quantification of metabolites (Figure 3) and the compound assignments table (Table S1) was also reported. In the case of some compounds, only a partial assignment was obtained due to low concentration of the compound. However, the partial assignment included diagnostic signals that allowed the identification of the reported compounds. Glucose and malic acid turned out to be the sugar and the organic acid, respectively, present in major amounts.

### 3.5. Preparation of P. domestica Fruit Extract without Sugar

In view of the high sugar content (about 14% of the whole extract) of *P. domestica* subsp. syriaca fruit pulp crude extract, it was subjected to chemical precipitation of sugar contents by treatment with absolute ethanol, supported by ultra-freezing temperature. The percent extraction yield following sugar precipitation was 41.8%.

### 3.6. Effect of P. domestica Subsp. Syriaca Fruit Pulp Extract on Enzyme Activities

**Figure 4:**
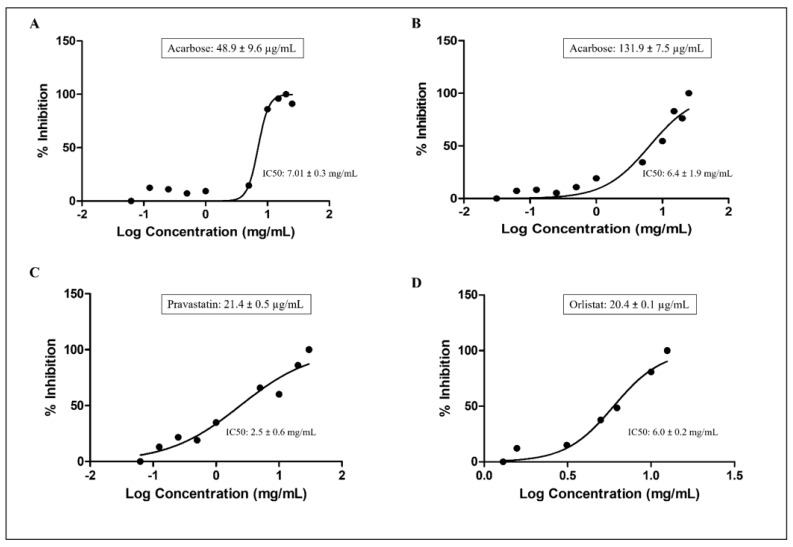
Enzyme inhibition activities and calculated IC50 values of *P. domestica* subsp. syriaca fruit pulp extract. α-amylases inhibition (**A**), α-glucosidase inhibition (**B**), HMG CoA reductase inhibition (**C**), Pancreatic lipase inhibition (**D**). Data expressed as mean ± SD. The IC50 values of the fruit extract against each enzyme were calculated using nonlinear regression analysis.

*P. domestica* subsp. syriaca fruit pulp extract with reduced content of sugars was used for enzyme inhibition activities, with the aim of reducing the substances that may interfere with the enzyme inhibition activities of the vegetable extract. The inhibition of the enzyme activities performed by the extract at increasing concentration and the IC50 values for each enzyme, calculated with the nonlinear regression analysis, have been illustrated in Figure 4.

*P. domestica* subsp. syriaca fruit pulp extract inhibited α-amylase and α-glucosidase enzymes in a concentration-dependent manner, with an IC50 value of 7.01 mg/mL compared to acarbose, used as positive control (IC50: 48.9 µg/mL) (Figure 4A), and IC50 value of 6.4 mg/mL compared to acarbose (IC50: 131.9 µg/mL) (Figure 4B), respectively. As far as HMG-CoA reductase is concerned, *P. domestica* subsp. syriaca fruit extract inhibited HMG-CoA reductase enzyme in a concentration-dependent manner with an IC50 value of 2.5 mg/mL, while the reference inhibitor pravastatin inhibited HMG-CoA reductase with an IC50 value of 21.4 µg/mL (Figure 4C). Finally, regarding pancreatic lipase, the results suggest that *P. domestica* subsp. syriaca fruit pulp extract inhibited this enzyme in a concentration-dependent manner with an IC50 value of 6.0 mg/mL compared to reference inhibitor orlistat (IC50 value: 20.4 µg/mL) (Figure 4D). Overall, the mean IC50 values of the fruit extract were found to be significantly different from the values obtained from the positive controls (*p* < 0.05).

### 3.7. In Vitro Anti-Inflammatory Effects of P. domestica Subsp. Syriaca Fruit Pulp Extract

**Figure 5:**
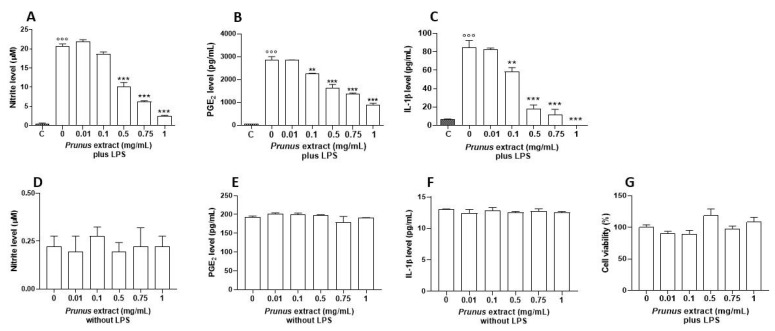
Effect of the *P. domestica* subsp. syriaca fruit pulp extract on LPS-induced nitrite, IL-1β and PGE_2_ production. J774 cells were pretreated for 2 h with increasing concentrations of the extract (‘0.01’. ‘0.1’. ‘0.5’. ‘0.75’. and ‘1’ mg/mL) and then stimulated with LPS (‘10’ µg/mL) for 24 h (plus LPS). Effects of extract were also evaluated in absence of LPS (without LPS). Unstimulated J774 cells acted as a negative control (**C**). Nitrites (**A**,**D**), stable end products of NO, were measured in the supernatants by the Griess reaction, whereas IL-1β (**B**,**E**) and PGE_2_ (**C**,**F**) were measured by ELISA. Cell viability was evaluated by the mitochondrial-dependent reduction of MTT to formazan (**G**). ◦◦◦ *p* < 0.001 vs. unstimulated cells (**C**), *p* < 0.001 vs. unstimulated cells (**C**), *** *p* < 0.001 and ** *p* < 0.01 vs. LPS alone.

In order to assess the anti-inflammatory proprieties of *P. domestica* subsp. syriaca fruit pulp extract, murine macrophage cell line J774 stimulated with LPS (10 µg/mL, 24 h), a well-known proinflammatory stimulus, was used. The anti-inflammatory activities were assessed by measuring the levels of proinflammatory mediators such as nitrites, PGE_2_ and IL-1β. Preincubation of J774 macrophages with *P. domestica* subsp. syriaca fruit pulp extract (2 h before LPS treatment) inhibited significantly and in a concentration-dependent manner (0.01, 0.1, 0.5, 0.75 and 1 mg/mL) the production of nitrite (IC50 0.46 mg/mL, Figure 5A), PGE_2_ (IC50 0.56 mg/mL, Figure 5B) and IL-1β (IC50 0.18 mg/mL, Figure 5C) induced by LPS, starting from the concentration of 0.1 mg/mL. No effects of P. domestica subsp. syriaca fruit pulp extract on proinflammatory mediator production were observed in unstimulated cells (without LPS) (Figure 5D–F). To rule out any alteration of cell viability, an MTT assay was performed and did not show any statistical reduction in cell viability after treatment with extract (Figure 5G).

## 4. Discussion

The World Health Organization (WHO) reports that approximately 650 million people live today with obesity, 422 million with T2DM and 1.13 billion people with hypertension [39,40,41]. Thus, metabolic disorders remain a prevalent and urgent concern in the healthcare field, which today are remedied through pharmacological approaches prescribed to target some specific pathogenetic mechanisms. This, in turn, is often accompanied by adverse effects and poor compliance by patients. [42]. Therefore, the study of new food supplement ingredients for the reduction of risk factors of MS is essential and finding new agents able to modulate the enzyme activities associated with carbohydrate/lipid digestion and cardiometabolic diseases is one of the essential targets in the prevention and treatment of cardiometabolic disorders. As evident by preclinical and clinical trials, *Prunus* species can improve energy homeostasis involving glucose and lipid metabolism, decrease inflammatory mediators, reduce lipid deposition, and modulate gut microbiota, and thus can reverse metabolic dysregulation states [43]. In this study, we explored the ability of a hydroethanolic extract of *P. domestica* subsp. syriaca (Mirabelle plum) fruit pulp to inhibit the activity of various enzymes associated with cardiometabolic disorders. Initially, fruit skin and pulp of two different *P. domestica* varieties (Common plum and Mirabelle plum), extracted with different concentrations of ethanolic solution, were evaluated for TPC, and the Mirabelle plum extract showing the highest TPC was selected for enzyme inhibition assays and evaluation of in vitro anti-inflammatory activity. Chemical profiling of *P. domestica* subsp. syriaca fruit pulp extract was evaluated using a multimethodological approach based on the application of new technologies, consisting of untargeted NMR spectroscopy and untargeted UHPLC-HRMS, which favors a holistic approach as opposed to the traditional reductionist methods, allowing us to overcome the concept of identifying one compound responsible for the obtained biological effect, and to ascribe the bioactivity to the whole phytocomplex [44]. The results suggested the presence of hydroxycinnamic acids (p-coumaroylquinic acid isomers and feruloylquinic acid derivatives), which resulted to be the most represented polyphenols followed by flavanols (catechin, epicatechin, procyanidins), flavonols (rutinoside and rhamnoside derivatives of quercetin), organic acids (quinic acid, citric acid, and malic acid), and carbohydrates (xylose, glucose, and sucrose). The carbohydrate quantitative analysis showed that glucose (106.6 mg/g dry weight of extract) is the main saccharide found in the fruit extract, in agreement with USDA Food Composition Databases, followed by sucrose (31.6 mg/g dry weight of extract) and xylose (0.6 mg/g dry weight of extract). Sugars of the fruit extract were at least in part chemically precipitated with ethanol before proceeding with the enzyme inhibition and the anti-inflammatory assays to remove the constituents that may interfere with the biological activities of the vegetable extract. The inhibition of α-amylase and α-glucosidase enzymes are important to reduce the digestion of complex carbohydrates and in turn the absorption of glucose, with the aim to normalize the blood glucose level both in subjects with mild hyperglycemia and in diabetic patients, to support glucose-lowering medication [19]. Acarbose is a pharmacologic drug currently employed in the treatment of subjects with diabetes due to its potential to inhibit α-amylase and α-glucosidase enzymes, thus reducing carbohydrate digestion, slowing down the absorption of carbohydrates, and decreasing postprandial insulin secretion, in addition to stimulating glucagon-like peptide (GLP-1) release [45]. The extract of *P. domestica* subsp. syriaca fruit pulp showed inhibitory activity against α-amylase and α-glucosidase enzymes with IC50 values of 7.01 mg/mL and 6.4 mg/mL, respectively. While evaluating antidiabetic activity of novel smoothies from selected *Prunus* fruits, Nowicka et al. demonstrated that anthocyanin and flavonol content have the highest impact on α-glucosidase enzyme, whereas flavanols may have the potential to inhibit α-amylase [46]. Some studies also reported that inhibition of α-glucosidase may be associated with the content of hydroxycinnamic acid derivatives such as ferulic acid or *p*-coumaric acids [47]. The researchers indicated that flavonols can interact with hydroxycinnamic acids or anthocyanins, which may increase the inhibition of α-glucosidase [48]. It has also been suggested that procyanidins-rich fruits are effective α-amylase inhibitors, possibly by the formation of enzyme-tannin complexes resulting in the prevention of the enzyme from interacting with starch [48]. The literature shows an inhibition of α-amylase and α-glucosidase enzymes with numerous botanical extracts, including *Elateriospermum tapos* Blume [49], *Xylopia parviflora* Spruce, *Monodora myristica* (Gaertn.) Dunal, *Tetrapleura tetraptera* (Schum. & Thonn.) Taub., *Dichrostachys glomerata* (Forssk.) Chiov., *Aframomum melegueta* K.Schum., *Aframomum citratum* (C.Pereira) K.Schum [34] and *Adansonia digitata* L. [33]. Different *Prunus* fruits (including Common European plum, ‘vlaškača’, damson plum, white damson, purple-leaf cherry plum, white cherry plum, red cherry plum, sweet cherry, sweet cherry-wild type, sour cherry, steppe cherry, mahaleb cheery, blackthorn, and peach) extracted with 50% ethanol exhibited inhibition of α-amylase (IC50 value range: 1.11–136.23 mg/mL) and α-glucosidase (IC50 value range: 0.41–28.44 mg/mL) [50]. *Prunus* species in general showed a greater affinity towards α-glucosidase enzyme compared to α-amylase [50]. Altogether, these data suggest that inhibition of the enzymes involved in the digestion of carbohydrates by vegetable extracts may have promising potential in the management of glucose metabolism disorders. Statins are effective lipid-lowering agents, widely used as a first-line therapy in the atherosclerotic CVDs, which are known to competitively inhibit HMG-CoA reductase enzyme (rate-limiting enzyme of cholesterol synthesis) [51]. Considering the same approach of decreasing cholesterol synthesis, *P. domestica* subsp. syriaca fruit pulp extract was tested against the HMG-CoA reductase activity, showing an IC50 value of 2.5 mg/mL. The mean IC50 value calculated for the fruit extract was significantly higher from the reference statin (pravastatin). Susilowati et al. (2020) performed in silico analysis while evaluating antihyperlipidemic effects of apple peel extract, which showed the highest HMG CoA reductase inhibition by catechin, epicatechin, quercetin (aglycosidal form) and chlorogenic acid [52]. Several other vegetable extracts have already shown HMG-CoA reductase inhibitory activities, including, but not limited to *Basella alba* L. [53], *Syzygium polyanthum* (Wight) Walp. [54], *Ficus palmata* Forssk. [55], and *Amaranthus viridis* L. [56]. Inhibition of pancreatic lipase is a clinically validated approach in the treatment of obesity, as it reduces the hydrolysis of fats and decreases their absorption [57]. The Food and Drug Administration (FDA) approved orlistat in 1999 for the pharmacological management of obesity in conjunction with a reduced caloric diet, while in 2007 it was approved as an over-the-counter (OTC) agent for weight loss in overweight adults (18 years or older) [58]. Later, the FDA revised the label for orlistat by adding a new warning about severe liver injury, which has been reported rarely with this drug [59]. Following concerns about the possible cause of severe hepatic toxicities with orlistat, the European Medicines Agency completed a review for this medicine, where the Agency’s Committee for Medicinal Products for Human Use (CHMP) concluded that the benefits of orlistat continue to outweighs the risks, but also recommended that marketing authorizations should ensure that the safety information on rarely occurring liver injuries be provided on the product information of all orlistat-containing medicines [60]. As reported in the present study, *P. domestica* subsp. syriaca fruit pulp extract inhibited the pancreatic lipase enzyme with an IC50 value of 6.0 mg/mL, although this value was significantly higher than that determined for orlistat. Hydroxycinnamic acids and proanthocyanidins have proven efficacy against lipase activity [61,62]. The presence of hydroxyl groups in the molecule (more potent), methoxy groups (less potent) and position of hydroxyl groups in the phenolic ring could influence the activity of polyphenols in inhibiting lipase enzyme [61]. Moreover, flavan-3-ol esters showed a stronger lipase inhibition compared to non-esterified flavanols such as catechin and epicatechin [61]. Plant species of different families (*Vitis vinifera* L., *Rhus coriaria* L., *Origanum dayi* Post, *Quercus infectoria* G.Olivier, *Eucalyptus galbie*, *Rosa damascene*, and *Levisticum officinale* W.D.J.Koch) showed considerable inhibition of pancreatic lipase enzyme [63,64]. Nowicka et al. observed an inhibition of pancreatic lipase enzyme with *Prunus persica* L. Batsch fruits (different cultivars), with an IC50 value ranging from 0.07 to 2.06 mg/mL [65]. On the whole, although the values of IC50 are much higher than those found for drugs, it must be considered that the extract, being derived from a food commonly consumed in diet and having been deprived of sugars, should not show adverse effects unlike medicines, and therefore it can be taken in larger quantities and for very long periods. In vivo studies are needed to confirm inhibitory activity against the enzymes considered. Lifestyle modifications (including diet and exercise) and pharmacological agents (such as peroxisome proliferator-activated receptor (PPAR)-α agonists, angiotensin-converting enzyme (ACE) inhibitors and angiotensin receptor blockers (ARBs)) all target inflammation in various ways, and thus can reduce MS-associated complications. Ongoing research studies are uncovering inflammatory pathways (related to obesity, T2DM, and MS), which may be potential targets for novel preventive and treatment strategies with the aim of improving overall patient quality of life and reducing mortality, preferentially by preventing the adverse sequelae from MS [66]. *P. domestica* subsp. syriaca fruit pulp extract showed promising results against nitrite, IL-1β, and PGE_2_ levels in LPS-stimulated macrophages in a concentration-dependent manner, with an IC50 of 0.46, 0.18 and 0.56 mg/mL, respectively. These results are in agreement with those previously published [67,68], although our extract was more active. In particular, dried plum polyphenols significantly suppressed the production of NO and COX-2 in LPS-stimulated RAW 264.7 macrophages at the concentration of 100 and 100 mg/mL, respectively [68]. The higher anti-inflammatory effects of *P. domestica* subsp. syriaca fruit pulp extract could be due to the presence of polyphenolic compounds in different concentrations, mainly flavonoids (catechin and (+) epicatechin) and hydroxy cinnamic acids (chlorogenic acid and feruloyl-coumaroylquinic acid derivative). Each compound inhibited the production of proinflammatory mediators at different concentrations, and whether the anti-inflammatory effects of this prunus extract are due to additively or synergistically polyphenolic action is not known. For example, inhibitory effects of catechin [69] and chlorogenic acid [70] on various inflammatory mediators using LPS-stimulated RAW 264.7 macrophages were reported. In particular, chlorogenic acid completely inhibited NO production in LPS-stimulated macrophage RAW 264.7 cells at the concentration of 40 ug/mL [70]. A medicinal plant *Inonotus (I.) sanghuang* (rich in rutin, chlorogenic acid, isorhamnetin, quercetin, and quercitrin) improved insulin resistance and MS by reducing inflammation via modulation of the crosstalk between macrophages and adipocytes [71]. Grape powder extract (rich in quercetin-3-glucoside, catechin, epicatechin, rutin, gallic acid and resveratrol) showed to decrease LPS-stimulated inflammation in macrophages by affecting the gene expression of IL-6, IL-8, IL-1β and TNF-α, and in turn decreased insulin resistance [72]. *Sambucus nigra* L. fruit extract alleviated insulin resistance by suppressing the enhanced production of NO, TNF-α, IL-6, and PGE_2_, where the presence of cyanidin-based anthocyanins, flavan-3-ols, flavonols, and hydroxycinnamic acids were detected [73].

The main strength of this study is represented by the comprehensive characterization of the *P. domestica* extract by different and complementary technologies and the evaluation of some in vitro functional activities of this extract on mechanisms related to the pathophysiology of cardiometabolic diseases. A limitation of this study is that we did not evaluate additional specific aspects, such as modulation of triglyceride content or glucose uptake in appropriate cell models. The assessment of these and other related parameters will be the object of future research, in order to complete the functional characterization of *P. domestica* extracts and lay the scientific foundations for the placing on the market of new food supplements based on *P. domestica* extracts.

## 5. Conclusions

Nutraceuticals and functional foods are known to play an important role in the maintenance of human health and wellbeing through the prevention of chronic diseases. A substantial increase in the worldwide usage of vegetable extracts has been observed in recent decades, probably due to the increasing trend of consumer propensity towards preventive care through natural substances. Currently, more than 80% of world population is relying on the use of vegetable products for their primary health concerns [74]. The present investigation studied the potential nutraceutical benefits of *P. domestica* subsp. syriaca fruit pulp extract and found that it inhibited key enzymes involved in the metabolism of carbohydrates and lipids as well as in cholesterol synthesis, and attenuated LPS-stimulated release of proinflammatory mediators. The chemical characterization showed the presence of polyphenolic contents, which potentially justifies these biological properties. Taken together, these findings point towards a potential to modulate some molecular mechanisms involved in the pathophysiology of cardiometabolic diseases. Further studies using in vitro and in vivo models are required to better characterize these properties and their potential applications for human health.

## Figures and Tables

**Table 1:** Fruit samples and yield of extracts.

Prunus Variety	Common Name	Skin Color	Fruit Part Extracted	Ethanol (%)	Dry Extract (g/g) ^1^	Extraction Yield (%)
*P. domestica* subsp. domestica	Common plum	Purple	Skin	99	0.42	42.0
				70	0.39	39.0
				50	0.38	38.0
			Pulp	99	0.60	59.5
				70	0.63	63.0
				50	0.67	67.0
*P. domestica* subsp. syriaca	Mirabelle plum	Yellow	Skin	99	0.37	37.0
				70	0.41	41.0
				50	0.43	43.0
			Pulp	99	0.45	45.0
				70	0.46	46.0
				50	0.49	49.0

^1^ The weight of dry extract obtained in grams per gram of sample used for extraction.

**Table 2:** Total phenolic content and Trolox equivalent concentration of the extracts obtained from the two varieties of *P. domestica*.

Prunus Variety	Fruit Part Extracted (Ethanol %)	TPC (GAE/g on Dry Weight Basis)	Trolox Equivalent Concentration (µM/g on Dry Weight Basis)
*P. domestica* subsp. domestica	skin (99%)	9.1 ± 1.0	1282.4 ± 84.1 ^a^
	skin (70%)	12.8 ± 0.9 ^a^	1826.2 ± 216.4
	skin (50%)	11.0 ± 0.6 ^b^	1944.1 ± 138.1 ^b^
	pulp (99%)	7.2 ± 1.0	630.5 ± 44.1 ^a^
	pulp (70%)	11.3 ± 0.2 ^a^	1611.9 ± 289.5
	pulp (50%)	9.7 ± 0.2 ^b^	1290.7 ± 155.5 ^b^
*P. domestica* subsp. syriaca	skin (99%)	7.0 ± 0.2	708.0 ± 25.1
	skin (70%)	11.2 ± 1.4	1597.4 ± 88.2 ^c^
	skin (50%)	7.9 ± 0.8 ^c^	1602.1 ± 368.1
	pulp (99%)	6.5 ± 0.4	578.5 ± 53.5
	pulp (70%)	10.0 ± 0.9	727.7 ± 43.9 ^c^
	pulp (50%)	12.9 ± 1.7 ^c^	1119.4 ± 93.1

Data are expressed as mean ± SD (*n* = 3). The assigned values of different letters in a column show significant difference among the mean values (*p* < 0.05); TPC, Total phenolic content.

**Table 3:** Identified compounds in *P. domestica* subsp. syriaca fruit pulp extract according to the retention time (RT), compound, *m/z* and MS/MS, molecular formula, and mass accuracy, reported as part per million (ppm) error.

Peak	r_t_	Compound	[M-H]^-^	MS/MS	Molecular Formula	Error (ppm)
1	0.60	Citric acid	191.0227	111.0103; 173.0103	C_6_H_8_O_7_	1.57
2	3.12	Chlorogenic acid	353.0874	173.0489; 191.0576	C_16_H_18_O_9_	−1.13
3	4.68	Coumaroylquinic acid Isomer	337.0945	163.0417; 119.0558	C_16_H_18_O_8_	4.75
4	5.45	Catechin	289.0729	245.0816	C_15_H_14_O_6_	3.81
5	6.08	(+) Epicatechin dimer B type	577.1328	407.0787; 289.0728	C_30_H_26_O_12_	−4.16
6	6.50	Feruloylquinic acid	367.1053	193.0531; 134.0390	C_17_H_20_O_9_	4.90
7	6.70	Coumaroylquinic acid isomer	337.0928	163.0447; 191.0594	C_16_H_18_O_8_	1.19
8	7.20	Coumaroylquinic acid isomer	337.0952	173.0458; 163.0418	C_16_H_18_O_8_	2.30
9	8.12	(+) Epicatechin	289.0735	245.0816	C_15_H_14_O_6_	5.88
10	8.48	(+) Epicatechin trimer B type	865.1979	407.0790; 287.0569; 577.1344	C_45_H_38_O_18_	3.40
11	8.86	(+) Epicatechin dimer B type isomer	577.1344	407.0790; 289.0732	C_30_H_26_O_12_	−1.39
12	9.70	Quinic acid derivative	393.1777	149.0465; 191.0561	C_17_H_30_O_10_	2.80
13	10.50	Feruloyl-coumaroylquinic acid derivative	559.1665	337.0947; 193.0514	C_24_H_32_O_15_	−0.54
14	11.29	Feruloyl-coumaroylquinic acid derivative	559.1670	337.0949; 193.0510	C_24_H_32_O_15_	−0.50
15	12.19	Feruloyl-coumaroylquinic acid derivative	559.1677	337.0946; 193.0514	C_24_H_32_O_15_	1.61
16	12.32	(+) Epicatechin dimer B type isomer	577.1358	407.0831; 289.0742	C_30_H_26_O_12_	1.04
17	12.74	(+) Epicatechin B type trimer isomer	865.2015	407.0778; 287.0569; 577.1344; 543.0905	C_45_H_38_O_18_	3.47
18	13.20	Quercetin-rutinoside	609.1477	301.0351; 271.0254; 255.0320	C_27_H_30_O_16_	3.47
19	13.48	(+) Epicatechin A type trimer	863.1823	575.1180; 423.0711; 285.0393	C_45_H_36_O_18_	−0.20
20	14.04	(+) Epicatechin A type trimer isomer	863.1828	575.1180; 423.0711; 285.0393	C_45_H_36_O_18_	−0.12
21	14.82	(+) Epicatechin A type dimer	575.1197	423.0746; 285.0395	C_30_H_24_O_12_	1.22
22	15.75	Quercetin-rhamnoside	447.0924	301.0371; 255.	C_21_H_20_O_11_	−0.9
23	16.52	(+) Epicatechin A type dimer isomer	575.1187	423.0716; 285.0398	C_30_H_24_O_12_	−1.39

**Table 4:** Retention time (min) and peak area, expressed as percentage of total area of the identified compounds in *P. domestica* subsp. syriaca fruit pulp extract.

Peak	Compound	Retention Time	Area %
1	Citric acid	0.6	7.92
2	Chlorogenic acid	3.12	15.43
3	Coumaroylquinic acid Isomer	4.68	0.74
4	Catechin	5.45	8.61
5	(+) Epicatechin dimer B type	6.08	5.74
6	Feruloylquinic acid	6.5	0.21
7	Coumaroylquinic acid isomer	6.7	0.39
8	Coumaroylquinic acid isomer	7.2	2.42
9	(+) Epicatechin	8.12	4.54
10	(+) Epicatechin trimer B type	8.48	0.24
11	(+) Epicatechin dimer B type isomer	8.86	5.47
12	Quinic acid derivative	9.7	1.98
13	Feruloyl-coumaroylquinic acid derivative	10.5	3.01
14	Feruloyl-coumaroylquinic acid derivative	11.29	19.55
15	Feruloyl-coumaroylquinic acid derivative	12.19	1.49
16	Feruloyl-coumaroylquinic acid derivative	12.32	1.47
17	(+) Epicatechin dimer B type isomer	12.74	6.27
18	(+) Epicatechin B type trimer isomer	13.2	0.79
19	Quercetin-rutinoside	13.48	3.20
20	(+) Epicatechin A type trimer	14.04	4.05
21	(+) Epicatechin A type trimer isomer	14.82	1.81
22	(+) Epicatechin A type dimer	15.75	0.16
23	Quercetin-rhamnoside	16.52	4.50

**Table 5:** Compounds identified in the ^1^H NMR spectrum of *P. domestica* subsp. syriaca fruit pulp extract dissolved in phosphate buffer/D2O and the corresponding chemical shift signals (ppm) used in the integration process. The compound amounts in μg/mg of dry weight are also reported.

Compound	Chemical Shift (ppm) of Selected Resonances Used for Quantification	μg/mg Dry Weight
Quinic acid	1.88 (CH2-1)	7.50
Citric acid	2.54 (α,γ-CH)	0.84
Malic acid	4.30 (α-CH)	38.49
Xylose	5.20 (CH-1)	0.56
Glucose	5.25 (CH-1)	106.59
Sucrose	5.42 (CH-1)	31.59

## Supplementary Materials

The following are available online at https://www.mdpi.com/article/10.3390/nu14020340/s1, Table S1: Sugars and organic acids identified in the 600.17 MHz ^1^H spectra of P. domestica fruit pulp extracts. Asterisks indicate signals selected for integration.
