# Supplementary material for: Hydroethanolic Extract of Prunus domestica L.: Metabolite Profiling and In Vitro Modulation of Molecular Mechanisms Associated to Cardiometabolic Diseases
Source: Nutrients. 2022 Jan 14;14(2):340. doi: 10.3390/nu14020340 (PMC8778072; doi:10.3390/nu14020340)
Supplement: Supplementary file 1 [file nutrients-14-00340-s001.zip › nutrients-1514193-supplementary.pdf]

Table S1. Sugars and organic acids identified in the 600.17 MHz ^{1}H spectra of *P. domestica* fruit pulp extracts. Asterisks indicate signals selected for integration.

Compound	Assignment	1H (ppm)	Multiplicity [J(Hz)]	13C (ppm)
**Sugars**				
@-Glucose	CH-1	5.25*	d [3.6]	93.1
	CH-2	3.55		72.0
	CH-3	3.72		73.4
	CH-4	3.43		70.6
	CH-5	3.84		
	CH_2-6,6'	3.89; 3.75		61.7
@-Glucose	CH-1	4.66*	d [8.1]	97.0
	CH-2	3.25		75.1
	CH-3	3.49		76.8
	CH-4	3.43		70.4
	CH-5	3.49		76.9
	CH_2-6,6'	3.90; 3.81		61.7
Sucrose	CH-1 (Glucose)	5.42*	d [3.8]	93.1
	CH-2	3.58		72.0
	CH-3	3.78		73.4
	CH-4	3.49		70.2
	CH-3' (Fructose)	4.23	d [8.8]	77.5
	CH-4'	4.06		75.0
	CH-5'	3.90		82.5
	CH_2-6'	3.83		
α-Xylose	CH-1	5.20*	d [3.6]	
	CH-2	3.52		
β- Xylose	CH-1	4.59*	d [7.9]	
**Organic acids**				
Citric acid	α,γ-CH	2.54*	d [15.3]	46.7
	α',γ'-CH	2.70		46.7
	β-C			76.4
	1,5-COOH			180.1
	6-COOH			182.8
Malic acid	α-CH	4.30*	dd [10.0; 3.3]	71.4
	β-CH	2.67	dd [15.5; 3.3]	43.7
	β'-CH	2.39	dd [15.3; 10.0]	43.7
Quinic acid	C-1			78.1
	CH_2-2, 2′	1.88*; 2.07	dd [13.3; 11.0]; m	41.9
	CH-3	4.01	m	67.9
	CH-4			76.2
